# Ischemic Sigmoid Colon Presenting As Hemoperitoneum From an Avulsed Mesocolon During Colonoscopy: A Case Report and Literature Review

**DOI:** 10.7759/cureus.64418

**Published:** 2024-07-12

**Authors:** Mueen Megdadi, Segala Tita, Aqib Javed, Jamal Zahir, Thomas Kang

**Affiliations:** 1 Department of General Surgery, Ascension Saint Agnes Hospital, Baltimore, USA; 2 Department of Surgery, Ross University School of Medicine, Miramar, USA

**Keywords:** avulsed mesocolon, hemoperitoneum, colonoscopy, sigmoid colon, ischemic colon

## Abstract

Colonoscopy has proven efficacy for both screening and diagnostic purposes. Although the risk of complications during colonoscopy is low, it is not negligible. As such, we present the case of a 72-year-old male patient who presented with abdominal pain and positive peritoneal signs post-colonoscopy. He was found to have anemia and acute on chronic kidney disease. An abdominal CT scan found evidence of hemoperitoneum. Subsequently, he underwent a diagnostic laparoscopy converted into an open exploratory laparotomy to identify the source of bleeding, an avulsed mesocolon. A review of the literature regarding colonoscopy and associated complications is discussed, highlighting the importance of risk stratification to better treat patients and prognosticate outcomes.

## Introduction

Colonoscopy is utilized for the screening, diagnosis, and prevention of GI pathology. Commonly, colonoscopies are performed in patients with lower GI bleeding, screening for colorectal cancer (CRC), and identifying polyps and/or structural abnormalities associated with specific syndromes and medical diseases (e.g., Lynch syndrome, familial adenomatous polyposis, and inflammatory bowel diseases such as Crohn’s and ulcerative colitis) [1].

It is important to keep in mind that other screening modalities such as fecal immunochemical tests (sensitivity 0.74, specificity 0.94 for CRC), hemoccult fecal occult blood tests (sensitivity 0.50-0.75, specificity 0.96-0.98 for CRC), and CT colonography (sensitivity 0.89, specificity 0.94 adenomas ≥10 mm) are alternative modalities to evaluate and diagnose bowel pathology [1-4].

A colonoscopy increases the risk of colonic ischemia and shearing of the mesocolon [5]. Colonic ischemia is the main form of vascular injury to the GI tract and is characterized by the sudden onset of nausea, abdominal pain, and bloody diarrhea [5]. This is often viewed as multifactorial, occurring either via splanchnic circulation impairment, drugs used during endoscopy, insufflation/barotrauma, or advancement of the scope [6].

Herein, we report the case of a 72-year-old man who developed an ischemic sigmoid colon presenting as hemoperitoneum after colonoscopy. In the following report, we reviewed the statistics of serious harm and overall complication rates resulting in hospitalization, as well as the rate of colonoscopy-related perforations.

## Case presentation

Our patient is a 72-year-old male with a past medical history of chronic kidney disease stage 3 (baseline creatinine 2.8), hypertension, chronic obstructive pulmonary disease, and thoracic aortic aneurysm status post-thoracic endovascular aortic repair. His past surgical history includes right inguinal hernia repair and surgical repair of the mandible after a traumatic fracture.

Clinical findings and diagnostic assessment

The patient initially presented to our hospital with generalized weakness, low appetite, acute on chronic kidney disease (creatinine of 3.6 mg/dL from 2.8 mg/dL baseline), and anemia without any signs of upper or lower GI bleeding. The patient's vitals were stable at that time, and labs were significant for hemoglobin of 10.2 g/dL, which trended down to 9.5 g/dL after fluid resuscitation. Due to his anemia, there was a concern for an occult GI bleed, and gastroenterology was consulted and upper and lower endoscopies were completed (no CT imaging was obtained). The esophagogastroduodenoscopy revealed a non-obstructing Schatzki ring, and the colonoscopy showed no masses, large polyps, or obvious arteriovenous malformations but was notable for multiple diverticula throughout the colon.

**Figure 1:**
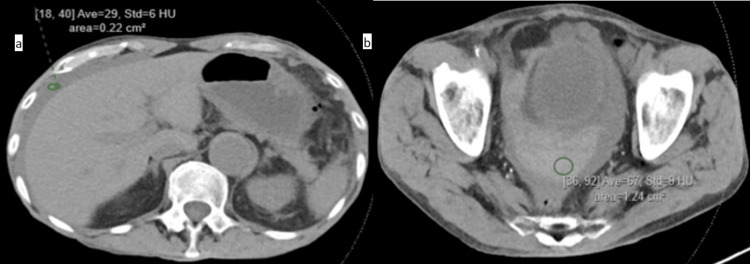
Axial view of non-contrast-enhanced CT of the abdomen and pelvis illustrating the hemoperitoneum (a) Free fluid anterolateral to the liver. (b) Pelvic-free fluid consistent with hemoperitoneum. Green circles signify the hemoperitoneum.

A few hours after the procedure, the patient started having diffuse abdominal pain, and hypotension (BP 84/40, HR 84) improved after a bolus of fluid. A complete lab panel was significant for a hemoglobin of 7.1 g/dL, down from 9.5 g/dL. On the exam, the patient had diffuse abdominal tenderness with positive peritoneal signs. To further investigate, a CT scan of the chest, abdomen, and pelvis without IV contrast was performed and revealed hemoperitoneum in the abdomen and pelvis, as shown below in Figure 1; its etiology was uncertain. There was no evidence of free intraperitoneal air or mechanical bowel obstruction.

**Figure 2:**
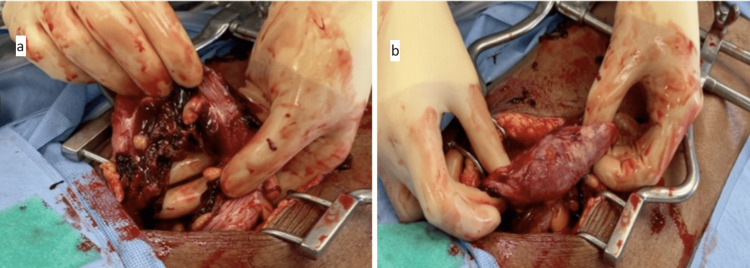
Intraoperative ischemic sigmoid colon visualized via exploratory laparotomy (a) Avulsed sigmoid mesentery. (b) Ischemic sigmoid colon.

Figure 2 illustrates the intraoperative findings during exploratory laparotomy after an unsuccessful initial diagnostic laparoscopy.

Surgical intervention

The patient was taken to the OR for a diagnostic laparoscopy, which was converted to an open exploratory laparotomy. This was followed by sigmoid resection with end colostomy, as seen in the illustration above.

Outcome and follow-up

In the initial postoperative period, the patient's hemoglobin stabilized, and the colostomy started functioning on day 1 postoperatively. Further, on postoperative day 1, he tolerated a regular diet and was discharged home in stable condition on postoperative day 3. On outpatient follow-up evaluation, he exhibited well-healed incisions and successful adaptation to a regular diet with a functioning colostomy. It is anticipated that colostomy reversal surgery will be scheduled in the foreseeable future.

## Discussion

Mesocolon avulsion typically occurs due to mechanical injury or shearing force, potentially resulting in colonic ischemia. Other causes of colonic ischemia include splanchnic circulation impairment, drugs used during endoscopy (e.g., narcotics, benzodiazepines (midazolam), and propofol), insufflation/barotrauma, or advancement of the scope by an inexperienced endoscopist placing undue pressure [5,6]. The variation of bleeding (intracolonic vs. extracolonic) was also analyzed. Intracolonic (luminal) bleeding is usually associated with polypectomy and rarely accompanies a diagnostic colonoscopy. It is also likely to be seen in other therapeutic maneuvers such as stricture dilation and endoscopic mucosal resection. Extracolonic bleeding, however, is rare, with the spleen being the most susceptible to injury, as indicated in Table 1 below [5].

**Table 1:** Types of extracolonic bleeding and the likelihood of occurrence extrapolated from the cited literature

Type of injury	Number of cases
Splenic injury	>100
Mesenteric tears	5
Retroperitoneal hemorrhage	2
Hepatic injury	1

The risks of colonic perforation also include injury from electrocautery during polypectomy, which is commonly performed in routine colonoscopy procedures [6]. Current statistics of occurrence from the mentioned complications of ischemic and avulsion based on the type of procedure being performed are as follows: screening colonoscopy (0.01-0.1%), anastomotic stricture dilation (0-6%), Crohn's disease stricture dilation (0-18%), stent placement (4%), colonic decompression tube placement (2%), and colonic endoscopic mucosal resection (0-5%) [6].

In an attempt to reduce unfavorable outcomes due to ischemia and colonic perforation, screening via CT colonography was also explored as a reliable test for CRC. It is mentioned that CT colonography appeared as likely as colonoscopy to detect lesions 10 mm or greater [5-7]. Although this test was less viable for smaller adenomas, the risk of perforation and other major complications was significantly lower than that of a traditional colonoscopy. Patients, however, were more likely to suffer from minor issues such as bacteremia [5-7].

The management of patients with suspected procedural complications, i.e., colonic ischemia or perforation, includes prompt abdominal radiography. Clinicians should be vigilant for post-procedural clinical signs (including but not limited to) nausea, bloody and non-bloody emesis, generalized severe abdominal tenderness, absence of bowel sounds, positive ascitic fluid exams, etc. [5].

In the review article by Kim et al., the incidence of adverse events such as colonic perforation and bleeding during colonoscopy was examined. They noted that blunt trauma caused the largest perforations, primarily in the rectosigmoid region, with a poor prognosis. Management strategies included using over-the-scope clips for larger perforations. Post-polypectomy bleeding was more common than in cases without polypectomy, categorized as immediate (within one day) or delayed (24 hours to 14 days), with a higher occurrence rate post-polypectomy (0.98%) compared to non-polypectomy (0.06%) (p<0.001) [7]. In the study by Weinstein et al., a case of sigmoid colon perforation during colonoscopy in a 67-year-old woman was detailed, revealing a 1.2 cm perforation via CT scan that necessitated a diagnostic laparotomy. Despite a history of chronic myelogenous leukemia, gastritis, and three C-sections, the patient had a normal postoperative follow-up. This study highlighted an iatrogenic perforation incidence of 0.032-0.15%, with higher rates in therapeutic procedures and most common at the sigmoid and rectosigmoid junctions (up to 70%) [8]. Unlike these cases, our patient experienced increasing abdominal pain a few hours post-colonoscopy without signs of perforation, nor did she warrant the need for an end colostomy, emphasizing the need for extreme vigilance during colonoscopy procedures.

A study by Langer et al. discussed the case of a 77-year-old woman who experienced a retroperitoneal hematoma and ischemic necrosis after a colonoscopy. Surgical intervention revealed an avulsed superior rectal artery and tight adhesions, leading to a Hartmann's procedure. The avulsed superior rectal artery likely occurred due to the tight S-shape of the sigmoid colon that the colonoscopy has to traverse, ultimately leading to iatrogenic damage and ischemic necrosis [9].

If there is hemodynamic instability and abdominal discomfort 24 hours post-colonoscopy, a clinician should have a high index of suspicion for splenic injury on his list of differential diagnoses. For example, in the study by Shankar and Rowe, a 47-year-old woman presented to the emergency department with left upper quadrant abdominal pain four hours post-colonoscopy with signs and symptoms of hypotensive shock and anemia. A CT abdomen revealed a grade IV splenic laceration, likely due to traction on the splenocolic ligament leading to capsular avulsion and subsequent splenic injury [10]. This patient was almost three decades younger than our patient, highlighting that colonoscopy-related intra-abdominal bleeding is also possible in younger patients.

McBride et al. reported a case of splenic injury post-colonoscopy in a 64-year-old woman with diverticulosis. She presented to the emergency department with LUQ abdominal pain, anemia, an elevated WBC count, and signs of hypotensive shock, all within one day after the colonoscopy. A CT scan revealed perisplenic hematoma and free fluid in the abdomen. She underwent an urgent laparotomy and splenectomy due to massive blood loss. The study highlighted that 60-70% of such patients required splenectomy and suggested preventive measures like avoiding supine positioning and applying external pressure to prevent splenic injury [11]. Again, this is an example of extracolonic bleeding (i.e., splenic injury) due to colonoscopy. Hence, it is imperative that gastroenterologists and practitioners conducting colonoscopies maintain a heightened level of awareness regarding this potential complication during the execution of such procedures.

Tagg et al. demonstrated a 59-year-old woman who developed mild abdominal pain and hemoperitoneum after a colonoscopy, with stable hemoglobin and no pneumoperitoneum or contrast extravasation on CT. The patient was conservatively managed and discharged after eight days, likely due to an injured mesenteric vein. This case demonstrates the feasibility of non-surgical management for hemoperitoneum post-colonoscopy [12]. Similarly, Choi et al. reported a 20-year-old male who experienced abdominal pain and was diagnosed with hemoperitoneum 12 hours post-colonoscopy. Laparoscopy revealed an isolated laceration of the mesocolon of the distal descending colon, underscoring the need to consider mesocolon avulsion in the differential diagnosis for post-colonoscopy intra-abdominal bleeding. This is most similar to our case in that the damage was iatrogenic [13]. Khosla et al. described a 48-year-old cirrhotic man who developed hemoperitoneum following colonoscopy due to external pressure, with imaging showing blood in the paracolic gutters. Managed conservatively, the patient likely ruptured a portal-caval collateral vessel, highlighting the risk of non-obstructive colonic ischemia if unnoticed [14]. Similar to our presenting case, the complication of the patient in Khosla et al., if gone unnoticed, could have resulted in non-obstructive colonic ischemia, which arguably would have warranted surgical intervention and possibly an end colostomy.

In the case report by Wu et al., a 51-year-old male experienced abdominal pain two days post-colonoscopic polypectomy, leading to a diagnosis of sigmoid mesenteric laceration. The patient, with a history of constipation, underwent successful mesocolon repair. The case highlighted the increased risk of avulsion and perforation in therapeutic procedures [15]. In contrast, Dehal and Tessier reported a 55-year-old man with Crohn’s disease who developed multiple types of pneumoperitoneum during a diagnostic colonoscopy, likely due to weakened tissue from prior biopsies. Surgical repair was necessary [16]. Unlike these cases, our patient did not have a therapeutic procedure or an underlying intestinal disease requiring polypectomy. Therefore, patients presenting similar to those in Dehal and Tessier should have a shorter delay to the diagnosis of colonic perforation/complications due to increased risk and higher suspicion.

In the case report by Yuan et al., a 70-year-old man with a history of colonic polypectomy developed abdominal pain five hours after an electrical polypectomy. A CT scan revealed extraluminal free air, and surgical exploration uncovered a 5 mm ileal perforation caused by electrocautery. The case underscores the need for careful biopsy and excision to prevent thermal damage to adjacent tissues [17]. In contrast, Ishii et al. described an 82-year-old woman with hypertension, atrial fibrillation, and chronic renal failure who developed non-occlusive mesenteric ischemia (NOMI) during bowel preparation. Imaging showed colonic obstruction, leading to a total colectomy and ileum resection. NOMI, induced by low blood volume from the bowel prep agents, has a high mortality rate [18]. Our patient, however, did not present with procedure-induced perforation or signs of obstruction, highlighting the critical role of clinical judgment and timely intervention in managing post-colonoscopy complications.

The case series by Lee et al. described two cases in which colonoscopy was employed for both screening and therapeutic purposes. In both cases, the patients developed ischemic colitis secondary to intestinal ischemia less than 24 hours after their colonoscopies. The proposed mechanism for this intestinal ischemia is air insufflation, which causes reduced intestinal blood flow. This is not surprising, as studies have shown that the colon receives less blood flow when compared to other areas of the alimentary tract [19]. It is possible that the etiology of the disease in our case was similar given that there was no evidence of free intraperitoneal air or mechanical bowel obstruction on repeat imaging, which confirmed the diagnosis of bowel ischemia.

Thus, it comes as no surprise that additional contemporary literature has also confirmed the persistent potential of colonic and extracolonic harm from colonoscopy, albeit not common [20-24]. Table 2 shows the summary of key articles discussing colonoscopy and colonic ischemia.

**Table 2:** Summary of key articles discussing colonoscopy and colonic ischemia

Author	Type of procedure (screening vs. diagnostic vs. therapeutic)	Site	Treatment (surgical vs. conservative)	Perforation (Y/N)	Proposed mechanism	Comments
Yuan et al., 2022 [17]	Therapeutic	Ileum (80 cm proximal from ileocecal valve)	Surgical	Y	Thermal injury	The patient had an electrical polypectomy in the transverse colon. Post-procedure a small perforation caused by thermal injury was diagnosed
Dehal and Tessier, 2014 [16]	Diagnostic	Terminal ileum	Surgical	Y	Biopsy-related injury	Case report. Patient with a history of Crohn’s disease and multiple biopsies at the terminal ileum. Presented with abdominal pain post-colonoscopy with pneumoperitoneum and pneumothorax
Ishii et al., 2019 [18]	Screening	Discontinuous from anus to ileum	Surgical	N	Low blood volume	Case report. Patient with bowel ischemia and necrosis during bowel prep
Lee et al., 2014 [19]	Case 1: therapeutic, case 2: screening	Case 1: ascending colon, case 2: distal transverse colon and proximal sigmoid colon	Conservative/supportive	N	Air insufflation	Case Series. No history of risk factors for colonic ischemia. Normal prognosis
Kim et al., 2019 [7]	Diagnostic vs. screening	Recto-sigmoid common	Endoscopic	NA	Mechanical trauma vs. thermal trauma vs. barotrauma	Literature review analyzing the incidence of various complications leading to colonic perforation during colonoscopy
Weinstein et al., 2023 [8]	Screening	Sigmoid colon	Surgical	Y	Mechanical (blunt) trauma	Case report and literature review analyzing methods of surgical intervention for perforation following colonoscopy
Sadalla et al., 2021 [5]	Diagnostic	Thrombosis of the superior mesenteric artery, ileocolic artery, and common hepatic artery	Reversible ischemia: conservative, irreversible ischemia: emergent surgery	N	NA	Case report/Literature review elaborating on mechanisms of colonic ischemia secondary to colonoscopy

## Conclusions

Colonic mesenteric tears are a rare but potentially life-threatening complication of a colonoscopy procedure. Awareness and a high index of suspicion are required to diagnose these patients. Practicing physicians must be able to identify subtle signs of clinical patient decline, including but not limited to worsening abdominal pain, new-onset GI bleeding, and anemic progression (decline of hemoglobin lab values, etc.). We hope that the provided case report and literature review prove useful to the world of gastroenterology and beyond, allowing for better prognostication of patients and thereby improving clinical outcomes.
